# Comparative transcriptome analysis of *Peromyscus leucopus* and C3H mice infected with the Lyme disease pathogen

**DOI:** 10.3389/fcimb.2023.1115350

**Published:** 2023-04-11

**Authors:** Alhussien M. Gaber, Igor Mandric, Caroline Nitirahardjo, Helen Piontkivska, Andrew E. Hillhouse, David W. Threadgill, Alex Zelikovsky, Artem S. Rogovskyy

**Affiliations:** ^1^ Department of Veterinary Pathobiology, School of Veterinary Medicine and Biomedical Sciences, Texas A&M University, College Station, TX, United States; ^2^ Department of Computer Science, Georgia State University, Atlanta, GA, United States; ^3^ Department of Biological Sciences, and School of Biomedical Sciences, Kent State University, Kent, OH, United States; ^4^ Brain Health Research Institute, Kent State University, Kent, OH, United States; ^5^ Texas A&M Institute for Genomics Sciences and Society, Texas A&M University, College Station, TX, United States; ^6^ Department of Molecular and Cellular Medicine, Texas A&M University Health Science Center, Texas A&M University, College Station, TX, United States

**Keywords:** Lyme borreliosis, *Borreliella burgdorferi*, *Borrelia*, *Peromyscus leucopus*, C3H mice, differentially expressed genes

## Abstract

Lyme disease (LD), the most prevalent tick-borne disease of humans in the Northern Hemisphere, is caused by the spirochetal bacterium of *Borreliella burgdorferi* (*Bb*) sensu lato complex. In nature, *Bb* spirochetes are continuously transmitted between *Ixodes* ticks and mammalian or avian reservoir hosts. *Peromyscus leucopus* mice are considered the primary mammalian reservoir of *Bb* in the United States. Earlier studies demonstrated that experimentally infected *P. leucopus* mice do not develop disease. In contrast, C3H mice, a widely used laboratory strain of *Mus musculus* in the LD field, develop severe Lyme arthritis. To date, the exact tolerance mechanism of *P. leucopus* mice to *Bb*-induced infection remains unknown. To address this knowledge gap, the present study has compared spleen transcriptomes of *P. leucopus* and C3H/HeJ mice infected with *Bb* strain 297 with those of their respective uninfected controls. Overall, the data showed that the spleen transcriptome of *Bb*-infected *P. leucopus* mice was much more quiescent compared to that of the infected C3H mice. To date, the current investigation is one of the few that have examined the transcriptome response of natural reservoir hosts to *Borreliella* infection. Although the experimental design of this study significantly differed from those of two previous investigations, the collective results of the current and published studies have consistently demonstrated very limited transcriptomic responses of different reservoir hosts to the persistent infection of LD pathogens.

**Importance:**

The bacterium *Borreliella burgdorferi* (*Bb*) causes Lyme disease, which is one of the emerging and highly debilitating human diseases in countries of the Northern Hemisphere. In nature, *Bb* spirochetes are maintained between hard ticks of *Ixodes* spp. and mammals or birds. In the United States, the white-footed mouse, *Peromyscus leucopus*, is one of the main *Bb* reservoirs. In contrast to humans and laboratory mice (e.g., C3H mice), white-footed mice rarely develop clinical signs (disease) despite being (persistently) infected with *Bb*. How the white-footed mouse tolerates *Bb* infection is the question that the present study has attempted to address. Comparisons of genetic responses between *Bb*-infected and uninfected mice demonstrated that, during a long-term *Bb* infection, C3H mice reacted much stronger, whereas *P. leucopus* mice were relatively unresponsive.

## Introduction

Lyme disease (LD) is the most prevalent tick-borne disease in North America and Europe with an estimate of ~476,000 annual cases in the United States (U.S.) alone (Mead, 2015; Kugeler et al., 2021). LD is caused by some members of *Borreliella burgdorferi* (*Bb*) sensu lato complex, which is divided into more than 20 genospecies. At least three genospecies (*Borreliella afzelii*, *Bb* sensu stricto*, Borreliella garinii*) have the capacity to cause LD in humans (Baranton et al., 1992; Canica et al., 1993; Kurtenbach et al., 2006). The horizontal transmission of LD is mediated by hard ticks of *Ixodes* spp., which acquire spirochetes from *Bb*-infected vertebrate carriers such as rodents and birds (Piesman and Gern, 2004; Barbour and Gupta, 2021). In the U.S., the white-footed mouse, *Peromyscus leucopus*, is considered the main mammalian reservoir of *Bb* spirochetes (Levine et al., 1985; Donahue et al., 1987). Clinical signs of LD patients are often flu-like symptoms that can be followed by arthritis and neurological or cardiac abnormalities (Smith et al., 2011; Radolf et al., 2021).

When *Bb* infects mammals, innate immune effectors such as toll-like receptor (TLR) 2 and myeloid differentiation antigen 88 (MyD88) become fully engaged (Hirschfeld et al., 1999; Wooten et al., 2002a; Wooten et al., 2002b; Bolz et al., 2004). MyD88 subsequently activates a toll/IL-1 receptor-domain-containing adapter-inducing interferon-β (TRIF) signaling (Petnicki-Ocwieja et al., 2013). Moreover, *Bb* outer surface lipoproteins lead to activation of nuclear factor kappa-light-chain-enhancer (NF-κB) (Wooten et al., 1996; Ebnet et al., 1997). This cascade leads to secretion of pro-inflammatory cytokines and chemokines for neutrophils, monocytes, and lymphocytes during LD infection in mice (Wooten and Weis, 2001; Verhaegh et al., 2017; Bockenstedt et al., 2021). The mounting immune response is also accompanied by production of interferons (IFN), IFNα and IFNβ (type I INF), and IFNγ in response to *Bb* antigens (Ganapamo et al., 2000; Crandall et al., 2006; Miller et al., 2008). As a result of the activated innate immune system, the acquired humoral immune response is developed, and this leads to lowering the spirochetal burden in various tissues (Barthold et al., 1997; McKisic and Barthold, 2000). In LD patients, Th1 lymphocytes and mononuclear cells mediate B lymphocyte production of neutralizing anti-*Bb* antibodies such as IgG1 and IgG3, which induce opsonization and activate complement defense mechanisms (Hechemy et al., 1988; Bockenstedt et al., 2021).

Similar to LD human patients, some laboratory mouse strains develop arthritis and carditis upon LD infection (Barthold et al., 1990; Armstrong et al., 1992; Brown and Reiner, 1999). The degree of susceptibility to *Bb*-induced arthritis in mice is predetermined by a number of factors including inter-strain genetic variability and the fitness variation of *Bb* strains (Barthold et al., 1992; Ma et al., 1998; Hanincová et al., 2008; Lin et al., 2014). The different capacity of mouse strains to regulate the localized inflammatory response is a major factor for the arthritis development (Ma et al., 1998). By using C3H/He mice, which develop severe Lyme arthritis (Barthold et al., 1990; Barthold et al., 1992), it was demonstrated that IFNα and IFNβ have an important role during an early LD infection. The upregulation of the interferon responsive genes contributes to the pathogenesis of LD arthritis in C3H/He mice (Crandall et al., 2006; Miller et al., 2008). Type I INF response can also be associated with differential regulation of genes that are involved in tissue repair processes (Crandall et al., 2006; Lochhead et al., 2021). In contrast to C3H strains, C57BL/6 mice, which are deficient in an intense interferon response to *Bb* infection (Crandall et al., 2006), develop only mild Lyme arthritis (Barthold et al., 1990; Crandall et al., 2006). In addition to type I IFN, interleukin 10 (IL-10), an anti-inflammatory cytokine, has a crucial role in the regulation of arthritis severity (Sonderegger et al., 2012). C57BL/6 mice lacking IL-10 exhibit more severe arthritis upon LD infection as opposed to their *Bb*-infected wild-type C57BL/6 controls (Brown et al., 1999).

In contrast to C3H mice, previous studies demonstrated that experimentally *Bb*-infected *P. leucopus* mice do not develop disease (Schwanz et al., 2011). Both *Bb*-infected and uninfected control *P. leucopus* mice were shown to have similar white blood cell counts and wheel-running activity (Schwanz et al., 2011). The resistance of persistently *Bb*-infected *P. leucopus* mice to the disease was also shown to be age-dependent since only infant mice could develop carditis and multifocal arthritis upon *Bb* infection (Moody et al., 1994). Of note, however, the tolerance of white-footed mice to *Bb*-induced disease is not absolute. A previous study demonstrated that a fraction (e.g., ~7-10%) of wild-caught *P. leucopus* population had clinical signs of LD (Burgess et al., 1990). The lack of detectable LD in the majority of *Bb*-infected *P. leucopus* population can potentially be explained by a long-term co-existence between the host and the pathogen. The outcome of this host-pathogen adaptation is likely to have created a balance between the strong but non-sterilizing immune response of reservoir hosts (Schwan et al., 1989; Baum et al., 2012; Cook and Barbour, 2015; Rogovskyy et al., 2015) and the persistent presence of *Bb* spirochetes. Unfortunately, to date, the exact factors and mechanism of tolerance or susceptibility of *P. leucopus* mice to *Bb*-induced disease are unknown. In the present study, we have explored infection-induced changes in gene expression to understand how *P. leucopus* mice are able to tolerate *Bb* infection. For that, we compared responses to *Bb* infection between *P. leucopus* and *Mus musculus* (C3H/HeJ, referred to here as C3H) mice at the transcriptomic level. The overall data showed that the spleen transcriptome of *P. leucopus* mice was much more quiescent compared to that of the C3H mice.

## Materials and methods

### Bacterial strain

*Borreliella burgdorferi* strain 297 was a generous gift of Troy Bankhead. Spirochetes were grown in liquid Barbour-Stoenner-Kelly medium supplemented with 6% rabbit serum (referred to here as BSK-II; Gemini Bio-Products, CA, USA) and incubated at 35°C under 2% CO_2_.

### Mouse experiment

Six males of *P. leucopus* mice acquired from The Peromyscus Genetic Stock Center (the University of South Carolina, SC, USA) and 6 males of C3H/HeJ (C3H) mice purchased from The Jackson Laboratory (Bar Harbor, ME, USA) were split into 4 groups of three mice each. After an adaptation period, each of 3 *P. leucopus* (5-8 weeks of age) and 3 C3H (4-6 weeks of age) mice was subcutaneously (s.c.) inoculated in the shoulder region with 100-μl inoculum containing ~1.0 x10^5^ cells of *B. burgdorferi* 297. The remaining 6 animals (uninfected controls) that were within the respective age range were s.c. inoculated with 100 μl of sterile saline.

To confirm the infection, ~50 μl of blood samples taken *via* maxillary bleed from the 6 *Bb*-inoculated mice at day 7 postinoculation (pi) were cultured in BSK-II at 35°C under 2% CO_2_. At day 70 pi, ear pinnae, bladder, tibiotarsal joint, and heart tissues were harvested from both infected and uninfected groups of mice and cultured in BSK-II as described (Rogovskyy et al., 2016). Dark-field microscopy was utilized on a weekly basis over a four-week period to examine tissue cultures for the presence or absence of viable spirochetes (**Table S1**). At day 70 pi, spleens were also harvested from the 12 mice. All spleens were individually preserved in Invitrogen RNAlater stabilization solution (Thermo Fisher Scientific, MA, USA) and stored at - 80°C until RNA extraction.

### RNA extraction, library preparation, and sequencing

Total RNA from spleen tissues was isolated by using QIAGEN RNeasy Mini Kit (QIAGEN, CA, USA) according to manufacturer’s instruction. The quality and concentration of the RNA samples were determined by using Agilent Tapestation 2200 system on the RNA screen tape (Agilent Technologies, CA, USA) and Qubit Broad Range flurometric assay (Thermo Fisher Scientific, MA, USA), respectively. All RNA samples were normalized to 80 ng/μL for input into the Illumina TruSeq Stranded mRNA LS library preparation kit (Illumina, CA, USA). Individual libraries were constructed and barcoded according to the manufacturer’s protocol (Illumina). Sequencing library quality was assessed *via* Agilent TapeStation 2200 D1000 DNA screen tape (Agilent Technologies) showing a final library size of approximately 270 bp and were quantified with the Qubit High Sensitivity dsDNA assay (Thermo Fisher Scientific). All libraries were normalized and pooled in equimolar concentration for sequencing on an Illumina NextSeq 500 75 cycle High Output kit to generate approximately 400 million 75 base-pair, single-end sequencing reads (approximately 33 million reads per sample). All raw data was uploaded to Illumina BaseSpace (basespace.illumina.com) for FASTQ generation and demultiplexing of sequencing reads for their respective samples. All sequences were deposited to the GenBank Sequence Read Archive (SRA) and their SRA accession numbers are as follows: SAMN32740077, SAMN32740078, SAMN32740079, SAMN32740080, SAMN32740081, SAMN32740082, SAMN32740083, SAMN32740084, SAMN32740085, SAMN32740086, SAMN32740087, SAMN32740088.

### Reads mapping

Single reads were trimmed and mapped by STAR (version 020201) (Dobin et al., 2013) to *Peromyscus leucopus* (white-footed mouse) draft genome assembly (UCI_PerLeu_2.1; BioProject PRJNA533285 https://www.ncbi.nlm.nih.gov/assembly/GCF_004664715.2) (Long et al., 2019), and to *Mus musculus* C3H/HeJ strain (C3H_HeJ_v1; BioProject PRJNA310854; https://www.ncbi.nlm.nih.gov/assembly/GCA_001632575.1/). To facilitate functional annotation and pathways analyses, once *Peromyscus* reads were mapped to the UCI_PerLeu_2.1 assembly, the respective gene IDs were converted to mouse mm38 assembly IDs (https://www.ncbi.nlm.nih.gov/assembly/GCF_000001635.20/) using TBLASTN because the latter had more extensive functional and gene ontology annotations as the most frequently used mouse reference genome. The identified genes were also cross-referenced with the human genome assembly GRCh38.p13 using Ensembl BioMart tools (http://ensembl.org/biomart/martview). BAM files were combined for each mouse (in other words, each sample was represented by a single BAM file), and expression of each gene was inferred by HTSeq-count (Putri et al., 2022) followed by DESeq2 analysis (Love et al., 2014b). Transcript counts and R code for differential gene expression analysis are available at https://github.com/RNAdetective/Bb_Pleu-C3H_study.

### Identification and pathway analyses of differentially expressed genes

Differential gene expression analysis was performed using DESeq2 (Love et al., 2014b). The P values were adjusted by the Benjamini–Hochberg method (Benjamini and Hochberg, 1995). A corrected P value (padj) of 0.05 and 0.1 and log2 fold change (FC) of 2 and 1.5 were set as the strict and relaxed thresholds for differentially expressed genes (DEGs), respectively. To better understand the biological significance of identified DEGs, functional annotation of DEGs, and their interaction relationships were examined using STRING database analysis (string-db.org) (von Mering et al., 2005), which considered both direct physical interactions as well as indirect interactions through participation in the same pathways. The functional enrichment analyses of DEGs were carried out using the relaxed cutoffs, where a medium confidence score of 0.4 was used. Their connection degree and correlation were shown in the interaction network. Additional functional annotation and pathway enrichment analyses were conducted using the Reactome and InnateDB (Sidiropoulos et al., 2017; Fabregat et al., 2018; Jassal et al., 2020). The latter is a specialized database specifically tailored toward capturing interactions linked with the innate immune response (Breuer et al., 2013).

## Results

### Differentially expressed genes identified in the spleen transcriptome of *Bb*-infected *P. leucopus* and C3H mice

When only the strict cut-off values were considered (padj 0.05/2 FC), 34 DEGs were identified in the spleen transcriptome of *Bb*-infected *P. leucopus* mice relative to their uninfected control group. Out of the 34 DEGs, 12 and 22 genes were found to be up- and down-regulated, respectively (**Tables S2**, **S3**). Additional 47 DEGs were detected using relaxed cut-off values (padj 0.1/1.5 FC). Thus, a total of 35 (**Table S2**) and 46 (**Table S3**) genes were up- and down-regulated in *Bb*-infected *P. leucopus* mice, respectively. For *Bb*-infected C3H mice, 421 DEGs, which included 159 up-regulated and 262 down-regulated genes, were identified when relaxed cut-off values were considered. Of the 421 DEGs, 92 genes had strict cut-off values with 39 up-regulated and 53 down-regulated DEGs (**Tables S4**, **S5**).

### Pathway analyses of differentially expressed genes identified in the spleen transcriptome of *Bb*-infected *P. leucopus* mice by the Reactome and InnateDB

To better understand functional changes associated with the *Bb* infection in *P. leucopus* mice, the genes that were homologous between *P. leucopus* and the reference mouse mm38 genome were identified using BLASTN, because the latter had more comprehensive functional annotations than what was available for *P. leucopus*. Thus, a total of 53 (15 up-regulated and 38 down-regulated) DEGs identified in *Bb*-infected *P. leucopus* mice that also had homologs in the mouse genome were subjected to the Reactome and InnateDB pathway analyses in order to identify whether these genes shared common functional annotations and/or pathways (Breuer et al., 2013; Sidiropoulos et al., 2017; Fabregat et al., 2018; Jassal et al., 2020).

The data showed that the top 5 significant pathways detected through the Reactome were associated with the following: erythrocyte uptake of oxygen and release of carbon dioxide, erythrocyte uptake of carbon dioxide and release of oxygen, O_2_/CO_2_ exchange in erythrocytes, hydroxycarboxylic acid-binding receptors and auto-degradation of Cdh1 by Cdh1:APC/C (ST3). Moreover, the pathway overrepresentation analysis in the InnateDB (Breuer et al., 2013) identified the following top 5 overrepresented pathways (a corrected P value of < 0.05): hepatitis C, protein processing in endoplasmic reticulum, adipocytokine signaling pathway, peroxisome proliferator-activated receptor (PPAR) signaling pathway, and TLR signaling pathway.

### Pathway analyses of differentially expressed genes identified in the spleen transcriptome of *Bb*-infected C3H mice by the Reactome and InnateDB

A total of 159 up-regulated and 262 down-regulated DEGs detected in *Bb*-infected C3H mice were also subjected to the Reactome pathway and InnateDB network analyses. The top 5 significant pathways identified for the up-regulated genes *via* the Reactome were associated with cell cycle, cell cycle check points, activation of ATM- and Rad3-related (ATR) kinase in response to replication stress, cell cycle miotic, and activation of the pre-replicative complex. The InnateDB analysis of the up-regulated genes resulted in identification of the top 5 significant pathways associated with cell cycle, cell cycle mitotics, G1/S-specific transcription, G2/M checkpoints, and activation of ATR kinase in response to replication stress.

Pathways associated with platelet activation, signaling and aggregation, nitric oxide stimulation of guanylate cyclase, hemostasis, platelet adhesion to exposed collagen, and formation of fibrin clot (clotting cascade) were detected by the Reactome analysis for the down-regulated DEGs. Lastly, the top 5 significant pathways identified for the down-regulated genes *via* the InnateDB network were related to hemostasis, nitric oxide stimulation of guanylate cyclase, platelet homeostasis, platelet activation, signaling and aggregation, and platelet adhesion to exposed collagen.

### Pathway analyses of differentially expressed genes shared by both *Bb*-infected mouse species

**Figure 1:**
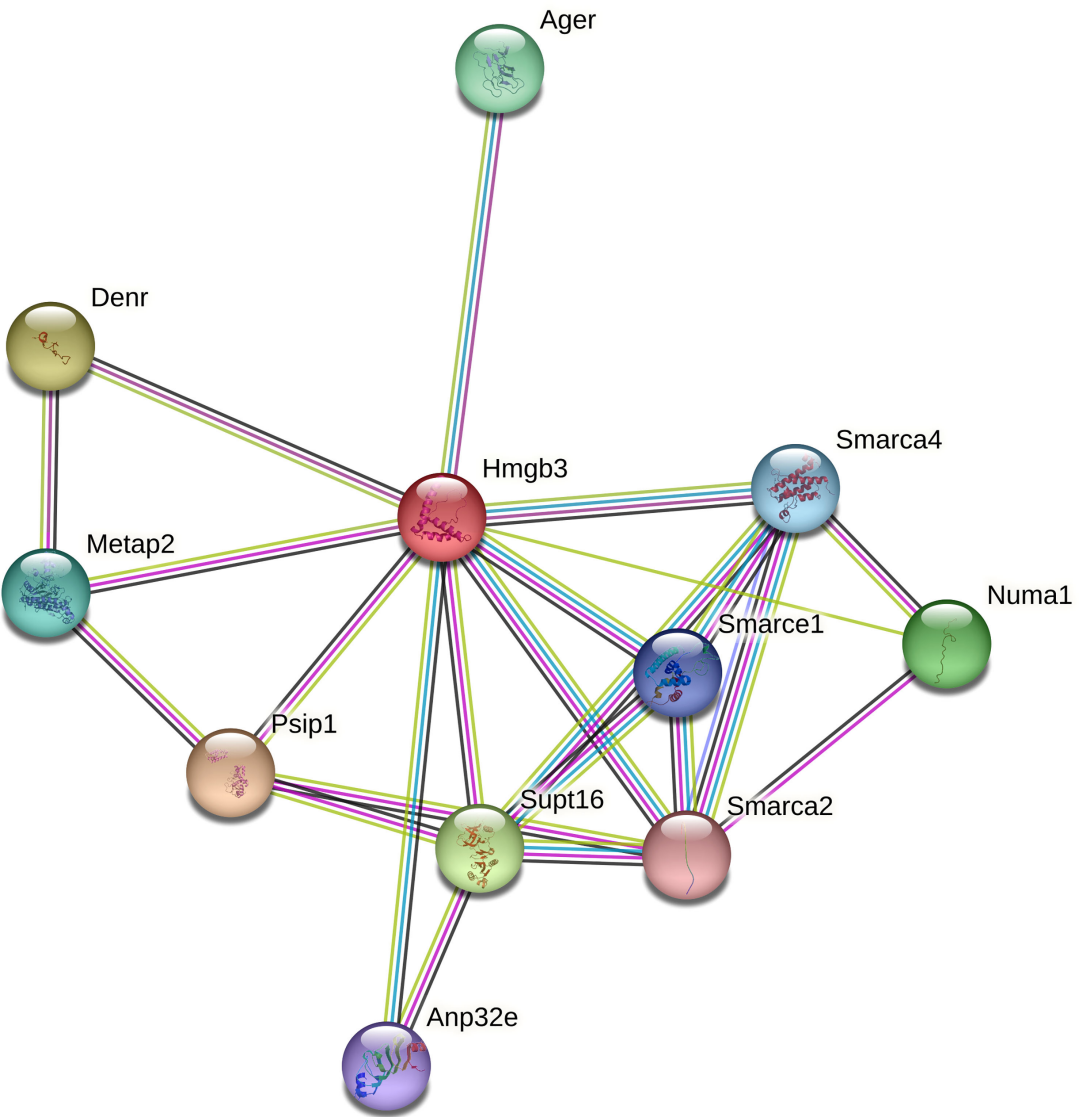
The protein-protein interaction network of HMGB3. The protein-protein interaction analyses were performed using the STRING database. Shown are HMGB3 (High Mobility Group Protein B3) and its top 10 protein interactors (listed clockwise): Ager (Advanced glycosylation end product-specific receptor), Smarca1, Smarca2, and Smarca4 (SWI/SNF related, matrix associated, actin dependent regulator of chromatin, subfamily A members 1, 2 and 4, respectively), Numa1 (Nuclear mitotic apparatus protein 1), Supt16 (Component of the FACT complex), Anp32e (Acidic leucine-rich nuclear phosphoprotein 32 family member E protein), PsiP1 (PC4 and SFRS1-interacting protein), Metap2 (Methionine aminopeptidase), and Denr (Density-regulated protein). Different line colors represent different types of interaction evidence between the proteins.

**Figure 2:**
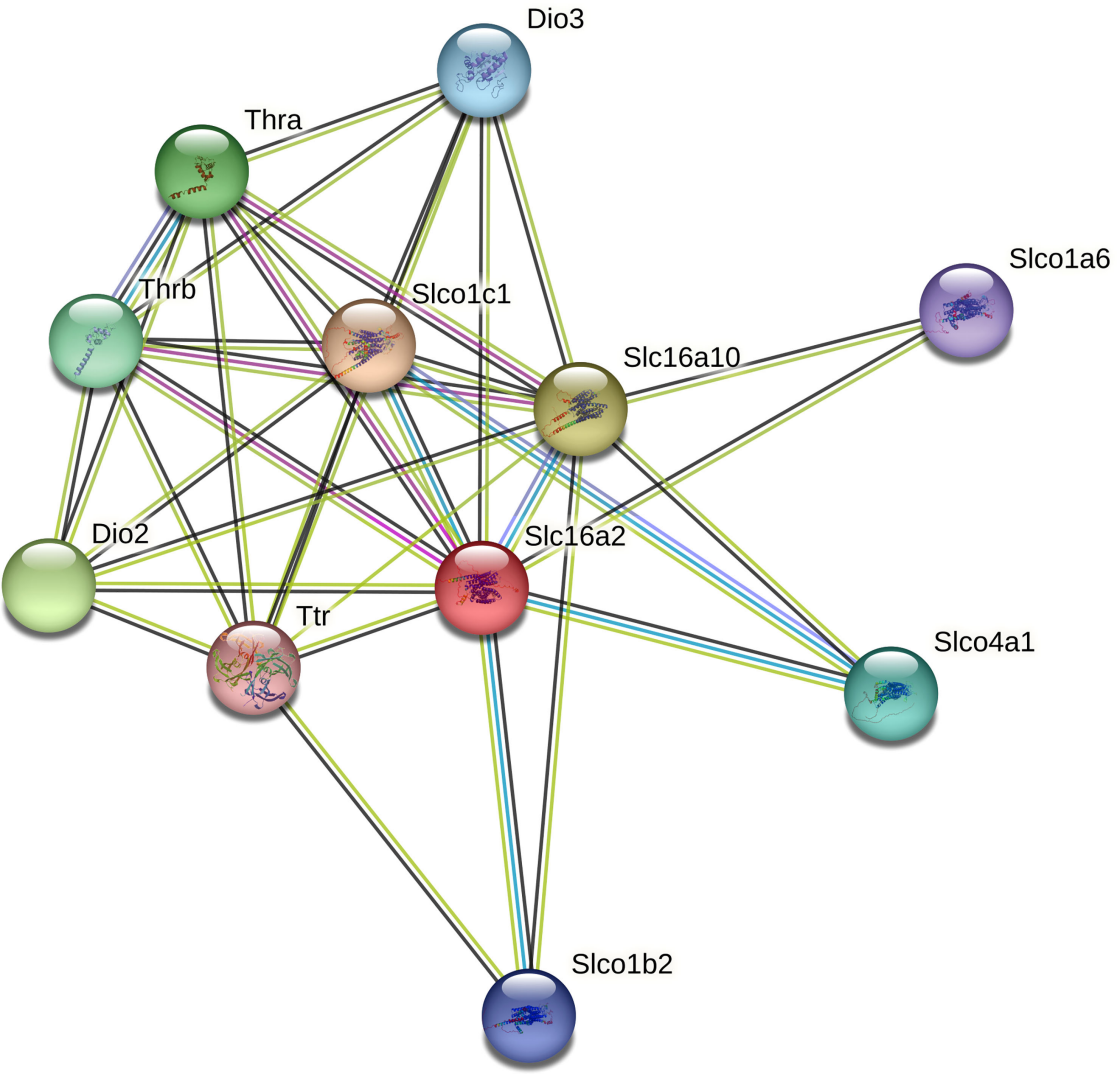
The protein-protein interaction network of Slc16a2. The protein-protein interaction analyses were performed using the STRING database. Shown are Slc16a2 (Solute carrier family 16 member 2 (Monocarboxylate transporter 8)) and its top 10 protein interactors (listed clockwise): Slco1c1 (Solute carrier organic anion transporter family member 1C1), Slc16a10 (Solute carrier family 16 member 10), Slco1a6 (Solute carrier organic anion transporter family member 1A6), Slco4a1 (Solute carrier organic anion transporter family member 4A1), Slco1b2 (Solute carrier organic anion transporter family member 1B2), Ttr (Transthyretin), Dio2 (Thyroxine 5-deiodinase type II), Thrb (Thyroid hormone receptors beta), Thra (Thyroid hormone receptors alpha), and Dio3 (Deiodinase, iodothyronine type III). Different line colors represent different types of interaction evidence between the proteins.

**Figure 3:**
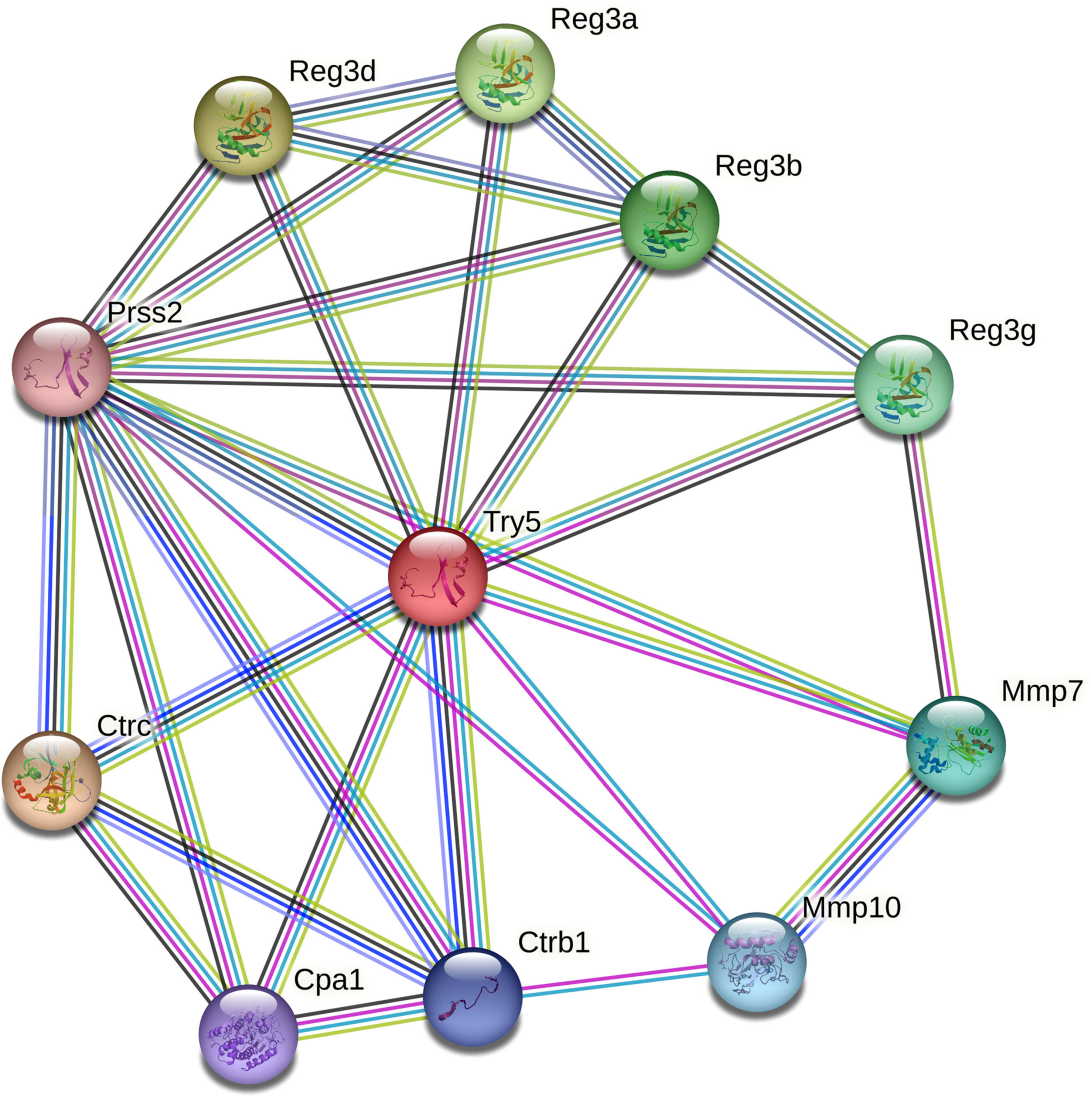
The protein-protein interaction network of Try5. The protein-protein interaction analyses were performed using the STRING database. Shown are Try5 (Trypsin 5) and its top 10 protein interactors (listed clockwise): Reg3a (Regenerating islet-derived 3 alpha), Reg3b (Regenerating islet-derived 3 beta), Reg3g (Regenerating islet-derived 3 gamma), Mmp7 (Matrix metallopeptidase 7), Mmp10 (Matrix metallopeptidase 10), Ctrb1 (Chymotrypsinogen B1), Cpa1 (Carboxypeptidase A1), Ctrc (Chymotrypsin C), Prss2 (Protease, serine 2), and Reg3d (Regenerating islet-derived 3 delta). Different line colors represent different types of interaction evidence between the proteins.

Only 3 DEGs were commonly identified for both *Bb*-infected mouse species. The HMGB3 gene encoding High Mobility Group Protein B3 was up- and down-regulated in infected C3H and *P. leucopus* mice, respectively. The top 10 interactors of the network identified by STRING for HMGB3 are shown in **Figure 1**. The Slc16a2 gene, which encodes solute carrier family 16 member 2 (monocarboxylate transporter 8) was down- and up-regulated in *Bb*-infected C3H and *P. leucopus* mice, respectively. The top 10 interactors of the Slc16a2 network are indicated in **Figure 2**. Lastly, the pathway analysis of the trypsin 5 (Try5)-encoding gene, which was up-regulated in both *Bb*-infected *P. leucopus* and C3H mice, identified the top 10 interactors of Try5 (**Figure 3**).

### Protein-protein interaction analyses by STRING

In order to better understand the biological functions of DEGs, protein-protein (PPI) interaction analyses were performed using the STRING database (Szklarczyk et al., 2019; Szklarczyk et al., 2021a; Szklarczyk et al., 2021b). The STRING PPI analysis was utilized to detect functionally associated proteins and their interconnections (Szklarczyk et al., 2019; Szklarczyk et al., 2021a; Szklarczyk et al., 2021b). Consequently, protein networks were constructed to show potential relationships of DEGs with different pathways.

**Figure 4:**
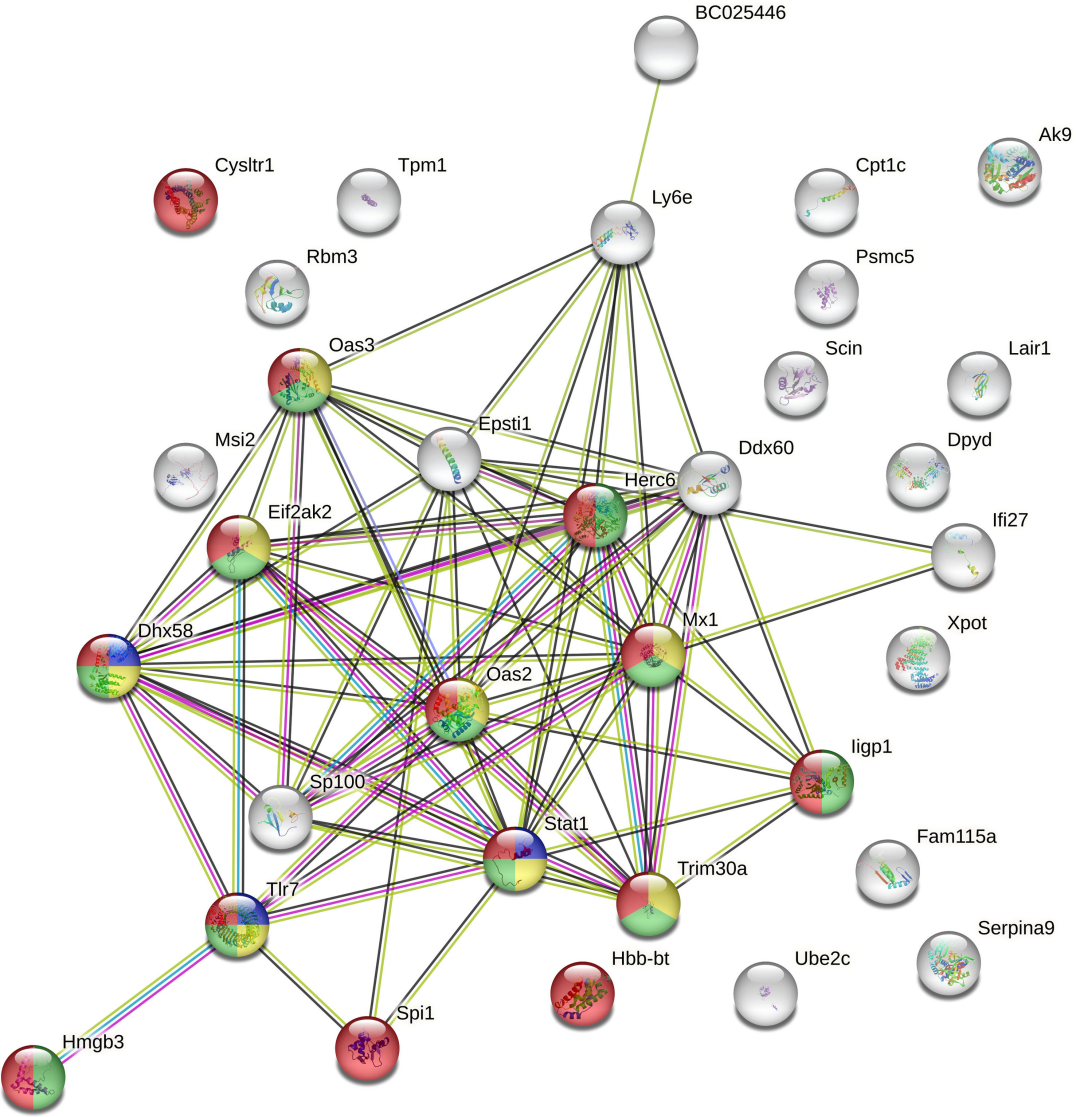
The protein-protein interaction network of down-regulated genes detected in *Borreliella burgdorferi*-infected *Peromyscus leucopus* mice. The protein-protein interaction analyses of 46 down-regulated genes identified in the spleen transcriptome of *B burgdorferi*-infected *P. leucopus* mice compared to their uninfected controls were performed using the STRING database. Colored are 14 proteins that are related to immune system process pathways (red), of which 11, 8, and 3 proteins are specifically associated with innate immune response (green), viral defense response (yellow), and positive regulation of type I INF production (blue), respectively.

When DEG categories were considered separately, the functional enrichment analysis of the 35 genes up-regulated in *Bb*-infected *P. leucopus* mice demonstrated only a few protein-protein interactions (15 nodes and 3 edges). In contrast, when the 46 down-regulated genes were analyzed, the network included a total of 32 nodes and 76 edges with 14 proteins related to immune system process pathways. Of the 14 proteins, 11, 8, and 3 proteins were found to be specifically associated with innate immune response, viral defense response, and positive regulation of type I INF production, respectively (**Figure 4**). The latter pathway also included genes encoding TLR7, signal transducer and activator of transcription 1 (STAT1), and ATP-dependent RNA helicase (DHX58).

When the entire set of 81 DEGs identified in *Bb*-infected *P. leucopus* mice was considered, the STRING analysis demonstrated significant enrichment of functional interactions among these genes (P value of < 0.1.0e-16), and overrepresentation of the following Gene Ontology (GO) molecular function terms: heterocyclic compound binding (25 genes), organic cyclic compound binding (25 genes), RNA binding (12 genes), double-stranded RNA binding (5 genes), and 2-5-oligoadenylate synthetase activity (2 genes) (**Figure S1**).

The STRING analysis of the 159 genes up-regulated in *Bb*-infected C3H mice identified 148 nodes and 1,551 edges in the network. In tissue expression and biological process terms, a total of 44 and 4 proteins were associated with immune system and B cell activation, respectively. Furthermore, in biological process term, 50 and 60 proteins were found to be related to pathways of cell cycle and cellular response to stimulus, respectively.

A total of 391 nodes and 2,095 edges were constructed by STRING for the 421 DEGs identified in *Bb*-infected C3H mice. In biological process term, some DEGs were enriched in the blood coagulation pathway (n=18). Out of 10 genes, whose proteins were associated with the regulation of blood coagulation, 9 and 1 genes were down- and up-regulated, respectively. The up-regulated gene encodes chymotrypsin-like elastase family member 2A (Cela2a). A total of 9 genes were associated with the platelet activation pathway, of which 8 and 1 genes were down- and up-regulated, respectively. The up-regulated gene encodes CD40 ligand (Cd40lg). When down-regulated genes were only considered (n=262), a total of 245 nodes and 407 edges were constructed by STRING with identification of 38 homeostasis-associated proteins *via* the Reactome (**Figure S2**). In biological process term, the STRING analysis showed that down-regulated genes were also enriched in wound healing (n=22), regulation of wound healing (n=11), response to stimulus (n=136), and response to stress (n=64).

## Discussion

In the present investigation, comparative transcriptomic analyses of spleens harvested from *Bb*-infected and uninfected *P. leucopus* mice have been performed. The spleen was selected because it is one of the major lymphoid organs that together with the bone marrow contains most immune cells (Zhao et al., 2012; Bronte and Pittet, 2013). The experimental design also included the laboratory “control” mouse strain – *Bb*-infected and uninfected C3H mice. To understand how *P. leucopus* mice are able to tolerate *Bb* infection, the transcriptome data generated from spleens of infected and uninfected mice were analyzed within each animal species by three complementing bio-molecular tools. The overall data demonstrated that *Bb*-infected *P. leucopus* mice had five times lower number of DEGs (n=81) than infected C3H mice (n=421). Out of the 81 DEGs, approximately 57% (n=46) and 43% (n=35) of genes were found to be down- and up-regulated, respectively. As opposed to the 46 down-regulated genes, many of whom were associated with immune response, the 35 up-regulated genes were not related to any immune response pathways. The overall data demonstrated that the *Bb*-induced response of the spleen transcriptome was more quiescent in *P. leucopus* mice compared to that of C3H mice.

The present results are similar to the findings of a previous study, where the spleen transcriptome analyses of *B. afzelii*-infected and uninfected bank voles (*Myodes glareolus*) and yellow-necked mice (*Apodemus flavicollis*) were performed (Zhong et al., 2020). Although both animal species are considered natural reservoir hosts of *B. afzelii* in Europe (Zhong et al., 2019), *Borreliella*-infected bank voles have on average a magnitude higher spirochetal loads in their tissues (Raberg, 2012; Zhong et al., 2019). The comparative analyses between infected and uninfected animals within each species identified only 8 and 5 DEGs in *B. afzelii*-infected bank voles and yellow-necked mice, respectively (Zhong et al., 2020). Furthermore, similar to the current data, other study had also shown no significant involvement of inflammation or innate immunity genes, when transcriptomes of skin and blood samples taken at 5 weeks postinfection were compared between *Bb* strain Sh-2-82-infected and uninfected *P. leucopus* mice (Long et al., 2019). Overall, the current and previous results could collectively suggest that reservoir hosts of LD pathogens moderate the immune system to avoid development of exaggerated (immune) responses.

A few examples of immune related genes that, during the persistent *Bb* infection, were down-regulated in *P. leucopus* mice and remained unresponsive in C3H mice include TLR7-encoding gene, type I IFN-associated genes, and members of the lymphocyte antigen-6 (*Ly6*) gene family. TLR7 recognizes bacterial RNA and peptidoglycan, and becomes activated in response to bacterial exposure by dendritic cells (DCs) (Mancuso et al., 2009). It was shown that TLR7 mRNA became up-regulated in *Bb-*exposed human oligodendrocyte cell line (Parthasarathy and Philipp, 2018). Overall, activation of TLRs by *Bb* lipoproteins leads to the induction of type I IFN (Petzke et al., 2009; Love et al., 2014a; Lochhead et al., 2021), which in turn primes macrophages for producing proinflammatory cytokines (Bockenstedt et al., 2021).

Through the use of laboratory mouse strains of *Mus musculus*, it has been consistently demonstrated that the severity of LD arthritis is driven by type I IFN (Crandall et al., 2006; Miller et al., 2008). Early type I IFN induction also positively correlates with the *Bb* capacity to disseminate in C3H/HeJ mice (Petzke et al., 2016). Type I IFN signaling may also lead to the accumulation of naïve B lymphocytes in *Bb*-infected mice (Hastey et al., 2014). Surprisingly, the present study did not identify any DEGs associated with the interferon signaling pathways in *Bb*-infected C3H mice. The latter cannot be solely explained by the lack of a functional TLR4 pathway in this particular mouse strain (Poltorak et al., 1998), partly because the classic enterobacterium-type lipopolysaccharide (LPS) (Brubaker et al., 2015), one of TLR4 ligands, is not produced by *Bb* spirochetes (Takayama et al., 1987). In contrast, a number of types I IFN-associated genes (n=6) were down-regulated in *Bb*-infected *P. leucopus* mice. These findings are consistent with the previous study, which demonstrated that a set of genes involved in IFNα signaling was down-regulated in *B. afzelii*-infected bank voles and yellow-necked mice compared to their uninfected controls (Zhong et al., 2020). In addition to type I IFN-associated genes, two members of *Ly6* gene family were also found to be down-regulated in *Bb*-infected *P. leucopus* mice. The 6E and 6A-2/6E-1-like proteins are known to be expressed by different types of mouse and human immune cells (e.g., B and T lymphocytes, natural killer (NK) cells, monocytes, and DCs) and involved in regulation of activation, proliferation, and differentiation of T lymphocytes (Ortega et al., 1986; Toulon et al., 1988; Lee et al., 2013).

Notably, of the 421 DEGs identified in *Bb*-infected C3H mice, a number of genes (n=38) were associated with different aspects of hemostasis. In the blood coagulation pathway alone, there were a total of 19 down-regulated genes with some involved in the regulation of blood coagulation (n=10) and others associated with platelet activation (n=9). Similar results were obtained by an earlier study, where blood, spleen and liver transcriptomes of LPS-treated-treated *Mus musculus* (BALB/cAnNCrl, referred here as BALB/c) and *P. leucopus* mice were compared to their respective non-treated controls (Balderrama-Gutierrez et al., 2021). The previous data showed that it was only in the blood of LPS-treated BALB/c mice where DEGs associated with negative regulation of blood coagulation pathways were detected (Balderrama-Gutierrez et al., 2021). The present study also demonstrated that the Cela2a and Cd40lg genes were up-regulated only in *Bb*-infected C3H mice. Cela2a hydrolyzes elastin and reduces platelet hyperactivation (Esteghamat et al., 2019), and Cd40lg is involved in immunoglobulin class switching, activation of B cell and T cell proliferation, enhancement of IL10 production, and induction of NF-kappa-B production (Armitage et al., 1992; Borrow et al., 1996; Van Kooten and Banchereau, 1996). As opposed to *P. leucopus*, *Bb*-infected C3H mice had also an overabundance of up-regulated cell cycle genes (n=50), whose products are known to play an important role in the host immune response (e.g., macrophage expansion and antibody class switching) (Laphanuwat and Jirawatnotai, 2019).

Overall, there were only 3 DEGs that were commonly identified in both animal species when infected with *Bb* (**Table 1**). The HMGB3 gene was up- and down-regulated in *Bb*-infected C3H and *P. leucopus* mice, respectively. As multifunctional molecules, HMGB proteins are proposed to play a role in the nucleic-acid-mediated activation of innate immune responses (Yanai et al., 2009). Being part of the cytosolic receptor–IRF3/NF-kB signaling pathway (Yanai et al., 2009), HMGB proteins can also stimulate TLR2, which, through its engagement with *Bb* lipoproteins, initiates expression of NF-κB-dependent cytokines (Shin et al., 2008). Another example of “shared” DEGs includes the Slc16a2 gene, which encodes solute carrier family 16 member 2 (monocarboxylate transporter 8) in *P. leucopus* and C3H mice. This active and specific thyroid hormone transporter mediates cellular uptake and transmembrane transporter activity of thyroxine (T4), triiodothyronine (T3), reverse triiodothyronine (rT3) and diidothyronine (Friesema et al., 2003; Friesema et al., 2005). Thyroid hormones have an important role in activation of neutrophils, NK cells, DCs, and B and T lymphocytes during infection (Rubingh et al., 2020). The corresponding genes were up- and down-regulated in *Bb*-infected *P. leucopus* and C3H mice, respectively. Lastly, both mouse species had only one “shared” gene up-regulated in response to *Bb* infection. The Try5 gene encodes anionic trypsin-2-like protein and trypsin 5 in *P. leucopus* and C3H mice, respectively.

**Table 1:** A list of genes that are deferentially expressed in both *Borreliella burgdorferi*-infected *Peromyscus leucopus* and C3H mice.

	Deferentially expressed genes (and their products) in infected mice compared to their uninfected controls	
	*P. leucopus*	C3H
**Up-regulated gene(s) in both infected species**	LOC114688085 (Anionic trypsin-2-like)	
**Down-regulated gene(s) in both infected species**	Not detected	
**Gene(s) up-regulated in infected *P. leucopus* mice and down-regulated in infected C3H mice**	Slc16a2 (Solute carrier family 16 member 2 (Monocarboxylate transporter 8))	
**Gene(s) down-regulated in infected *P. leucopus* and up-regulated in infected C3H mice**	HMGB3 (High Mobility Group Protein B3)	

Some limitations of this study include small sample sizes and the use of male mice only. Another caveat is that spirochetal burdens were not determined in spleens of the infected mice to see if potentially higher bacterial loads in C3H mice would explain overall higher numbers of DEGs identified in the laboratory mice compared to *P. leucopus* mice. In addition to addressing the above limitations, future studies are also warranted to confirm the identified DEGs through other approaches (e.g., qRT-PCR, proteomics).

In summary, the present investigation is one of the few that have examined the transcriptome response of mammalian reservoir hosts to *Borreliella* infection. Although the experimental design of this study is different from the above-mentioned earlier investigations (e.g., reservoir host, *Borreliella* strain, time point of harvest, tissue type analyzed), the collective results of all studies have consistently demonstrated a very limited response of natural reservoir hosts to the persistent infection of LD pathogens. In order to decipher tolerance mechanism of *P. leucopus* and other reservoir hosts to *Bb* infection, future investigations that would pursue some of the data leads identified in the present and earlier studies are highly warranted.

## Data availability statement

The data presented in the study are deposited in the GenBank Sequence Read Archive (SRA) depository and the SRA accession numbers are SAMN32740077, SAMN32740078, SAMN32740079, SAMN32740080, SAMN32740081, SAMN32740082, SAMN32740083, SAMN32740084, SAMN32740085, SAMN32740086, SAMN32740087, SAMN32740088.

## Ethics statement

The animal study was reviewed and approved by the Institutional Animal Care and Use Committee of Texas A&M University.

## Author contributions

AG analyzed the data and produced the manuscript draft. AH performed sequencing and raw data assembly. IM and AZ were involved in the data analysis. CN and HP analyzed the data, contributed to the data visualization and writing of the manuscript. DT provided resources and contributed to the data analysis. AR concepted and developed the study, provided resources, oversaw the project, and wrote the manuscript. All authors contributed to the article and approved the submitted version.

## References

[B1] Armitage R. J. Fanslow W. C. Strockbine L. Sato T. A. Clifford K. N. Macduff B. M.. (1992). Molecular and biological characterization of a murine ligand for CD40. Nature 357 (6373), 80–82. doi: 10.1038/357080a0 1374165

[B2] Armstrong A. L. Barthold S. W. Persing D. H. Beck D. S. (1992). Carditis in Lyme disease susceptible and resistant strains of laboratory mice infected with *Borrelia burgdorferi* . Am. J. Trop. Med. Hyg. 47 (2), 249–258. doi: 10.4269/ajtmh.1992.47.249 1503192

[B3] Balderrama-Gutierrez G. Milovic A. Cook V. J. Islam M. N. Zhang Y. Kiaris H.. (2021). An infection-tolerant mammalian reservoir for several zoonotic agents broadly counters the inflammatory effects of endotoxin. mBio 12 (2). doi: 10.1128/mBio.00588-21 PMC8092257 33849979

[B4] Baranton G. Postic D. Saint Girons I. Boerlin P. Piffaretti J. C. Assous M.. (1992). Delineation of *Borrelia burgdorferi* sensu stricto, *Borrelia garinii* sp. nov., and group VS461 associated with Lyme borreliosis. Int. J. Syst. Bacteriol. 42 (3), 378–383. doi: 10.1099/00207713-42-3-378 1380285

[B5] Barbour A. G. Gupta R. S. (2021). The family *Borreliaceae* (Spirochaetales), a diverse group in two genera of tick-borne spirochetes of mammals, birds, and reptiles. J. Med. Entomol. 58 (4), 1513–1524. doi: 10.1093/jme/tjab055 33903910 PMC13395813

[B6] Barthold S. W. Beck D. S. Hansen G. M. Terwilliger G. A. Moody K. D. (1990). Lyme borreliosis in selected strains and ages of laboratory mice. J. Infect. Dis. 162 (1), 133–138. doi: 10.1093/infdis/162.1.133 2141344

[B7] Barthold S. W. Feng S. Bockenstedt L. K. Fikrig E. Feen K. (1997). Protective and arthritis-resolving activity in sera of mice infected with *Borrelia burgdorferi* . Clin. Infect. Dis. 25 Suppl 1, S9–17. doi: 10.1086/516166 9233658

[B8] Barthold S. W. Sidman C. L. Smith A. L. (1992). Lyme borreliosis in genetically resistant and susceptible mice with severe combined immunodeficiency. Am. J. Trop. Med. Hyg. 47 (5), 605–613. doi: 10.4269/ajtmh.1992.47.605 1449201

[B9] Baum E. Hue F. Barbour A. G. (2012). Experimental infections of the reservoir species *Peromyscus leucopus* with diverse strains of *Borrelia burgdorferi*, a Lyme disease agent. MBio 3 (6), e00434–e00412. doi: 10.1128/mBio.00434-12 23221801 PMC3517863

[B10] Benjamini Y. Hochberg Y. (1995). Controlling the false discovery rate: A practical and powerful approach to multiple testing. J. R. Statist. Soc. Ser. B 57, 289–300. doi: 10.2307/2346101

[B11] Bockenstedt L. K. Wooten R. M. Baumgarth N. (2021). Immune response to *Borrelia*: Lessons from Lyme disease spirochetes. Curr. Issues Mol. Biol. 42, 145–190. doi: 10.21775/cimb.042.145 33289684 PMC10842262

[B12] Bolz D. D. Sundsbak R. S. Ma Y. Akira S. Kirschning C. J. Zachary J. F.. (2004). MyD88 plays a unique role in host defense but not arthritis development in Lyme disease. J. Immunol. 173 (3), 2003–2010. doi: 10.4049/jimmunol.173.3.2003 15265935

[B13] Borrow P. Tishon A. Lee S. Xu J. Grewal I. S. Oldstone M. B.. (1996). CD40L-deficient mice show deficits in antiviral immunity and have an impaired memory CD8+ CTL response. J. Exp. Med. 183 (5), 2129–2142. doi: 10.1084/jem.183.5.2129 8642323 PMC2192549

[B14] Breuer K. Foroushani A. K. Laird M. R. Chen C. Sribnaia A. Lo R.. (2013). InnateDB: Systems biology of innate immunity and beyond-recent updates and continuing curation. Nucleic Acids Res. 41 (Database issue), D1228–D1233. doi: 10.1093/nar/gks1147 23180781 PMC3531080

[B15] Bronte V. Pittet M. J. (2013). The spleen in local and systemic regulation of immunity. Immunity 39 (5), 806–818. doi: 10.1016/j.immuni.2013.10.010 24238338 PMC3912742

[B16] Brown C. R. Reiner S. L. (1999). Experimental Lyme arthritis in the absence of interleukin-4 or gamma interferon. Infect. Immun. 67 (7), 3329–3333. doi: 10.1128/iai.67.7.3329-3333.1999 10377109 PMC116514

[B17] Brown J. P. Zachary J. F. Teuscher C. Weis J. J. Wooten R. M. (1999). Dual role of interleukin-10 in murine Lyme disease: Regulation of arthritis severity and host defense. Infect. Immun. 67 (10), 5142–5150. doi: 10.1128/iai.67.10.5142-5150.1999 10496888 PMC96863

[B18] Brubaker S. W. Bonham K. S. Zanoni I. Kagan J. C. (2015). Innate immune pattern recognition: A cell biological perspective. Annu. Rev. Immunol. 33, 257–290. doi: 10.1146/annurev-immunol-032414-112240 25581309 PMC5146691

[B19] Burgess E. C. French J. B. Jr. Gendron-Fitzpatrick A. (1990). Systemic disease in *Peromyscus leucopus* associated with *Borrelia burgdorferi* infection. Am. J. Trop. Med. Hyg. 42 (3), 254–259. doi: 10.4269/ajtmh.1990.42.254 2316794

[B20] Canica M. M. Nato F. du Merle L. Mazie J. C. Baranton G. Postic D. (1993). Monoclonal antibodies for identification of *Borrelia afzelii* sp. nov. associated with late cutaneous manifestations of Lyme borreliosis. Scand. J. Infect. Dis. 25 (4), 441–448. doi: 10.3109/00365549309008525 8248743

[B21] Cook V. Barbour A. G. (2015). Broad diversity of host responses of the white-footed mouse *Peromyscus leucopus* to *Borrelia* infection and antigens. Ticks Tick. Borne Dis. 6 (5), 549–558. doi: 10.1016/j.ttbdis.2015.04.009 26005106 PMC4504778

[B22] Crandall H. Dunn D. M. Ma Y. Wooten R. M. Zachary J. F. Weis J. H.. (2006). Gene expression profiling reveals unique pathways associated with differential severity of Lyme arthritis. J. Immunol. 177 (11), 7930–7942. doi: 10.4049/jimmunol.177.11.7930 17114465

[B23] Dobin A. Davis C. A. Schlesinger F. Drenkow J. Zaleski C. Jha S.. (2013). STAR: Ultrafast universal RNA-seq aligner. Bioinformatics 29 (1), 15–21. doi: 10.1093/bioinformatics/bts635 23104886 PMC3530905

[B24] Donahue J. G. Piesman J. Spielman A. (1987). Reservoir competence of white-footed mice for Lyme disease spirochetes. Am. J. Trop. Med. Hyg. 36 (1), 92–96. doi: 10.4269/ajtmh.1987.36.92 3812887

[B25] Ebnet K. Brown K. D. Siebenlist U. K. Simon M. M. Shaw S. (1997). *Borrelia burgdorferi* activates nuclear factor-kappa B and is a potent inducer of chemokine and adhesion molecule gene expression in endothelial cells and fibroblasts. J. Immunol. 158 (7), 3285–3292. doi: 10.4049/jimmunol.158.7.3285 9120285

[B26] Esteghamat F. Broughton J. S. Smith E. Cardone R. Tyagi T. Guerra M.. (2019). *CELA2A* mutations predispose to early-onset atherosclerosis and metabolic syndrome and affect plasma insulin and platelet activation. Nat. Genet. 51 (8), 1233–1243. doi: 10.1038/s41588-019-0470-3 31358993 PMC6675645

[B27] Fabregat A. Jupe S. Matthews L. Sidiropoulos K. Gillespie M. Garapati P.. (2018). The reactome pathway knowledgebase. Nucleic Acids Res. 46 (D1), D649–d655. doi: 10.1093/nar/gkx1132 29145629 PMC5753187

[B28] Friesema E. C. Ganguly S. Abdalla A. Manning Fox J. E. Halestrap A. P. Visser T. J. (2003). Identification of monocarboxylate transporter 8 as a specific thyroid hormone transporter. J. Biol. Chem. 278 (41), 40128–40135. doi: 10.1074/jbc.M300909200 12871948

[B29] Friesema E. C. Jansen J. Milici C. Visser T. J. (2005). Thyroid hormone transporters. Vitam. Horm. 70, 137–167. doi: 10.1016/s0083-6729(05)70005-4 15727804

[B30] Ganapamo F. Dennis V. A. Philipp M. T. (2000). Early induction of gamma interferon and interleukin-10 production in draining lymph nodes from mice infected with *Borrelia burgdorferi* . Infect. Immun. 68 (12), 7162–7165. doi: 10.1128/iai.68.12.7162-7165.2000 11083848 PMC97833

[B31] Hanincová K. Ogden N. H. Diuk-Wasser M. Pappas C. J. Iyer R. Fish D.. (2008). Fitness variation of *Borrelia burgdorfer*i sensu stricto strains in mice. Appl. Environ. Microbiol. 74 (1), 153–157. doi: 10.1128/aem.01567-07 17981941 PMC2223198

[B32] Hastey C. J. Ochoa J. Olsen K. J. Barthold S. W. Baumgarth N. (2014). MyD88- and TRIF-independent induction of type I interferon drives naive B cell accumulation but not loss of lymph node architecture in Lyme disease. Infect. Immun. 82 (4), 1548–1558. doi: 10.1128/iai.00969-13 24452685 PMC3993384

[B33] Hechemy K. E. Harris H. L. Duerr M. J. Benach J. L. Reimer C. B. (1988). Immunoglobulin G subclasses specific to *Borrelia burgdorferi* in patients with Lyme disease. Ann. N. Y. Acad. Sci. 539, 162–169. doi: 10.1111/j.1749-6632.1988.tb31849.x 3263825

[B34] Hirschfeld M. Kirschning C. J. Schwandner R. Wesche H. Weis J. H. Wooten R. M.. (1999). Cutting edge: Inflammatory signaling by *Borrelia burgdorferi* lipoproteins is mediated by toll-like receptor 2. J. Immunol. 163 (5), 2382–2386. doi: 10.4049/jimmunol.163.5.2382 10452971

[B35] Jassal B. Matthews L. Viteri G. Gong C. Lorente P. Fabregat A.. (2020). The reactome pathway knowledgebase. Nucleic Acids Res. 48 (D1), D498–d503. doi: 10.1093/nar/gkz1031 31691815 PMC7145712

[B36] Kugeler K. J. Schwartz A. M. Delorey M. J. Mead P. S. Hinckley A. F. (2021). Estimating the frequency of Lyme disease diagnoses, United States 2010-2018. Emerg. Infect. Dis. 27 (2), 616–619. doi: 10.3201/eid2702.202731 33496229 PMC7853543

[B37] Kurtenbach K. Hanincová K. Tsao J. I. Margos G. Fish D. Ogden N. H. (2006). Fundamental processes in the evolutionary ecology of Lyme borreliosis. Nat. Rev. Microbiol. 4 (9), 660–669. doi: 10.1038/nrmicro1475 16894341

[B38] Laphanuwat P. Jirawatnotai S. (2019). Immunomodulatory roles of cell cycle regulators. Front. Cell Dev. Biol. 7. doi: 10.3389/fcell.2019.00023 PMC6399147 30863749

[B39] Lee P. Y. Wang J. X. Parisini E. Dascher C. C. Nigrovic P. A. (2013). Ly6 family proteins in neutrophil biology. J. Leukoc. Biol. 94 (4), 585–594. doi: 10.1189/jlb.0113014 23543767

[B40] Levine J. F. Wilson M. L. Spielman A. (1985). Mice as reservoirs of the Lyme disease spirochete. Am. J. Trop. Med. Hyg. 34 (2), 355–360. doi: 10.4269/ajtmh.1985.34.355 3985277

[B41] Lin Y. P. Benoit V. Yang X. Martinez-Herranz R. Pal U. Leong J. M. (2014). Strain-specific variation of the decorin-binding adhesin DbpA influences the tissue tropism of the Lyme disease spirochete. PloS Pathog. 10 (7), e1004238. doi: 10.1371/journal.ppat.1004238 25079227 PMC4117581

[B42] Lochhead R. B. Strle K. Arvikar S. L. Weis J. J. Steere A. C. (2021). Lyme arthritis: Linking infection, inflammation and autoimmunity. Nat. Rev. Rheumatol. 17 (8), 449–461. doi: 10.1038/s41584-021-00648-5 34226730 PMC9488587

[B43] Long A. D. Baldwin-Brown J. Tao Y. Cook V. J. Balderrama-Gutierrez G. Corbett-Detig R.. (2019). The genome of *Peromyscus leucopus*, natural host for Lyme disease and other emerging infections. Sci. Adv. 5 (7), eaaw6441. doi: 10.1126/sciadv.aaw6441 31355335 PMC6656541

[B44] Love M. I. Huber W. Anders S. (2014b). Moderated estimation of fold change and dispersion for RNA-seq data with DESeq2. Genome Biol. 15 (12), 550. doi: 10.1186/s13059-014-0550-8 25516281 PMC4302049

[B45] Love A. C. Schwartz I. Petzke M. M. (2014a). *Borrelia burgdorferi* RNA induces type I and III interferons *via* toll-like receptor 7 and contributes to production of NF-κB-dependent cytokines. Infect. Immun. 82 (6), 2405–2416. doi: 10.1128/iai.01617-14 24664510 PMC4019181

[B46] Ma Y. Seiler K. P. Eichwald E. J. Weis J. H. Teuscher C. Weis J. J. (1998). Distinct characteristics of resistance to *Borrelia burgdorferi*-induced arthritis in C57BL/6N mice. Infect. Immun. 66 (1), 161–168. doi: 10.1128/iai.66.1.161-168.1998 9423853 PMC107872

[B47] Mancuso G. Gambuzza M. Midiri A. Biondo C. Papasergi S. Akira S.. (2009). Bacterial recognition by TLR7 in the lysosomes of conventional dendritic cells. Nat. Immunol. 10 (6), 587–594. doi: 10.1038/ni.1733 19430477

[B48] McKisic M. D. Barthold S. W. (2000). T-cell-independent responses to *Borrelia burgdorferi* are critical for protective immunity and resolution of Lyme disease. Infect. Immun. 68 (9), 5190–5197. doi: 10.1128/iai.68.9.5190-5197.2000 10948143 PMC101777

[B49] Mead P. S. (2015). Epidemiology of Lyme disease. Infect. Dis. Clin. North Am. 29 (2), 187–210. doi: 10.1016/j.idc.2015.02.010 25999219

[B50] Miller J. C. Ma Y. Bian J. Sheehan K. C. Zachary J. F. Weis J. H.. (2008). A critical role for type I IFN in arthritis development following *Borrelia burgdorferi* infection of mice. J. Immunol. 181 (12), 8492–8503. doi: 10.4049/jimmunol.181.12.8492 19050267 PMC3024833

[B51] Moody K. D. Terwilliger G. A. Hansen G. M. Barthold S. W. (1994). Experimental *Borrelia burgdorferi* infection in *Peromyscus leucopus* . J. Wildl. Dis. 30 (2), 155–161. doi: 10.7589/0090-3558-30.2.155 8028098

[B52] Ortega G. Korty P. E. Shevach E. M. Malek T. R. (1986). Role of Ly-6 in lymphocyte activation. I. Characterization of a monoclonal of a monoclonal antibody to a nonpolymorphic Ly-6 specificity. J. Immunol. 137 (10), 3240–3246. doi: 10.4049/jimmunol.137.10.3240 2430016

[B53] Parthasarathy G. Philipp M. T. (2018). Intracellular TLR7 is activated in human oligodendrocytes in response to *Borrelia burgdorferi* exposure. Neurosci. Lett. 671, 38–42. doi: 10.1016/j.neulet.2018.01.058 29408631 PMC5889718

[B54] Petnicki-Ocwieja T. Chung E. Acosta D. I. Ramos L. T. Shin O. S. Ghosh S.. (2013). TRIF mediates toll-like receptor 2-dependent inflammatory responses to *Borrelia burgdorferi* . Infect. Immun. 81 (2), 402–410. doi: 10.1128/iai.00890-12 23166161 PMC3553797

[B55] Petzke M. M. Brooks A. Krupna M. A. Mordue D. Schwartz I. (2009). Recognition of *Borrelia burgdorferi*, the Lyme disease spirochete, by TLR7 and TLR9 induces a type I IFN response by human immune cells. J. Immunol. 183 (8), 5279–5292. doi: 10.4049/jimmunol.0901390 19794067

[B56] Petzke M. M. Iyer R. Love A. C. Spieler Z. Brooks A. Schwartz I. (2016). *Borrelia burgdorferi* induces a type I interferon response during early stages of disseminated infection in mice. BMC Microbio.l 16, 29. doi: 10.1186/s12866-016-0644-4 PMC4784397 26957120

[B57] Piesman J. Gern L. (2004). Lyme borreliosis in Europe and north America. Parasitology 129 Suppl, S191–S220. doi: 10.1017/s0031182003004694 15938512

[B58] Poltorak A. He X. Smirnova I. Liu M. Y. Van Huffel C. Du X.. (1998). Defective LPS signaling in C3H/HeJ and C57BL/10ScCr mice: Mutations in *Tlr4* gene. Science 282 (5396), 2085–2088. doi: 10.1126/science.282.5396.2085 9851930

[B59] Putri G. H. Anders S. Pyl P. T. Pimanda J. E. Zanini F. (2022). Analysing high-throughput sequencing data in Python with HTSeq 2.0. Bioinformatics 38 (10), 2943–2945. doi: 10.1093/bioinformatics/btac166 35561197 PMC9113351

[B60] Raberg L. (2012). Infection intensity and infectivity of the tick-borne pathogen *Borrelia afzelii* . J. Evol. Biol. 25 (7), 1448–1453. doi: 10.1111/j.1420-9101.2012.02515.x 22536945

[B61] Radolf J. D. Strle K. Lemieux J. E. Strle F. (2021). Lyme disease in humans. Curr. Issues Mol. Biol. 42, 333–384. doi: 10.21775/cimb.042.333 33303701 PMC7946767

[B62] Rogovskyy A. S. Casselli T. Tourand Y. Jones C. R. Owen J. P. Mason K. L.. (2015). Evaluation of the importance of VlsE antigenic variation for the enzootic cycle of *Borrelia burgdorferi* . PloS One 10 (4), e0124268. doi: 10.1371/journal.pone.0124268 25893989 PMC4404307

[B63] Rogovskyy A. S. Gillis David C. Ionov Y. Gerasimov E. Zelikovsky A. Bäumler Andreas J. (2016). Antibody response to Lyme disease spirochetes in the context of VlsE-mediated immune evasion. Infect. Immun. 85 (1), e00890–e00816. doi: 10.1128/IAI.00890-16 27799330 PMC5203654

[B64] Rubingh J. van der Spek A. Fliers E. Boelen A. (2020). The role of thyroid hormone in the innate and adaptive immune response during infection. Compr. Physiol. 10 (4), 1277–1287. doi: 10.1002/cphy.c200003 32969509

[B65] Schwan T. G. Kime K. K. Schrumpf M. E. Coe J. E. Simpson W. J. (1989). Antibody response in white-footed mice (*Peromyscus leucopus*) experimentally infected with the Lyme disease spirochete (*Borrelia burgdorferi*). Infect. Immun. 57 (11), 3445–3451. doi: 10.1128/iai.57.11.3445-3451.1989 2807530 PMC259851

[B66] Schwanz L. E. Voordouw M. J. Brisson D. Ostfeld R. S. (2011). *Borrelia burgdorferi* has minimal impact on the Lyme disease reservoir host *Peromyscus leucopus* . Vector Borne Zoonotic Dis. 11 (2), 117–124. doi: 10.1089/vbz.2009.0215 20569016

[B67] Shin O. S. Isberg R. R. Akira S. Uematsu S. Behera A. K. Hu L. T. (2008). Distinct roles for MyD88 and toll-like receptors 2, 5, and 9 in phagocytosis of *Borrelia burgdorferi* and cytokine induction. Infect. Immun. 76 (6), 2341–2351. doi: 10.1128/iai.01600-07 18378636 PMC2423091

[B68] Sidiropoulos K. Viteri G. Sevilla C. Jupe S. Webber M. Orlic-Milacic M.. (2017). Reactome enhanced pathway visualization. Bioinformatics 33 (21), 3461–3467. doi: 10.1093/bioinformatics/btx441 29077811 PMC5860170

[B69] Smith B. G. Cruz A. I. Jr. Milewski M. D. Shapiro E. D. (2011). Lyme disease and the orthopaedic implications of Lyme arthritis. J. Am. Acad. Orthop. Surg. 19 (2), 91–100. doi: 10.5435/00124635-201102000-00004 21292932 PMC3656475

[B70] Sonderegger F. L. Ma Y. Maylor-Hagan H. Brewster J. Huang X. Spangrude G. J.. (2012). Localized production of IL-10 suppresses early inflammatory cell infiltration and subsequent development of IFN-γ-mediated Lyme arthritis. J. Immunol. 188 (3), 1381–1393. doi: 10.4049/jimmunol.1102359 22180617 PMC3262892

[B71] Szklarczyk D. Gable A. L. Lyon D. Junge A. Wyder S. Huerta-Cepas J.. (2019). STRING v11: Protein-protein association networks with increased coverage, supporting functional discovery in genome-wide experimental datasets. Nucleic Acids Res. 47 (D1), D607–d613. doi: 10.1093/nar/gky1131 30476243 PMC6323986

[B72] Szklarczyk D. Gable A. L. Nastou K. C. Lyon D. Kirsch R. Pyysalo S.. (2021a). Correction to ‘The STRING database in 2021: Customizable protein-protein networks, and functional characterization of user-uploaded gene/measurement sets’. Nucleic Acids Res. 49 (18), 10800. doi: 10.1093/nar/gkab835 34530444 PMC8501959

[B73] Szklarczyk D. Gable A. L. Nastou K. C. Lyon D. Kirsch R. Pyysalo S.. (2021b). The STRING database in 2021: Customizable protein-protein networks, and functional characterization of user-uploaded gene/measurement sets. Nucleic Acids Res. 49 (D1), D605–d612. doi: 10.1093/nar/gkaa1074 33237311 PMC7779004

[B74] Takayama K. Rothenberg R. J. Barbour A. G. (1987). Absence of lipopolysaccharide in the Lyme disease spirochete, *Borrelia burgdorferi* . Infect. Immun. 55 (9), 2311–2313. doi: 10.1128/iai.55.9.2311-2313.1987 3623705 PMC260699

[B75] Toulon M. Palfree R. G. Palfree S. Dumont F. J. Hämmerling U. (1988). Ly-6 A/E antigen of murine T cells is associated with a distinct pathway of activation. requirements for interferon and exogenous interleukin 2. Eur. J. Immunol. 18 (6), 937–942. doi: 10.1002/eji.1830180616 2454826

[B76] Van Kooten C. Banchereau J. (1996). CD40-CD40 ligand: A multifunctional receptor-ligand pair. Adv. Immunol. 61, 1–77. doi: 10.1016/s0065-2776(08)60865-2 8834494

[B77] Verhaegh D. Joosten L. A. B. Oosting M. (2017). The role of host immune cells and *Borrelia burgdorferi* antigens in the etiology of Lyme disease. Eur. Cytokine Netw. 28 (2), 70–84. doi: 10.1684/ecn.2017.0396 28840838

[B78] von Mering C. Jensen L. J. Snel B. Hooper S. D. Krupp M. Foglierini M.. (2005). STRING: Known and predicted protein-protein associations, integrated and transferred across organisms. Nucleic Acids Res. 33 (Database issue), D433–D437. doi: 10.1093/nar/gki005 15608232 PMC539959

[B79] Wooten R. M. Ma Y. Yoder R. A. Brown J. P. Weis J. H. Zachary J. F.. (2002a). Toll-like receptor 2 is required for innate, but not acquired, host defense to *Borrelia burgdorferi* . J. Immunol. 168 (1), 348–355. doi: 10.4049/jimmunol.168.1.348 11751980

[B80] Wooten R. M. Ma Y. Yoder R. A. Brown J. P. Weis J. H. Zachary J. F.. (2002b). Toll-like receptor 2 plays a pivotal role in host defense and inflammatory response to *Borrelia burgdorferi* . Vector Borne Zoonotic Dis. 2 (4), 275–278. doi: 10.1089/153036602321653860 12804169

[B81] Wooten R. M. Modur V. R. McIntyre T. M. Weis J. J. (1996). *Borrelia burgdorferi* outer membrane protein a induces nuclear translocation of nuclear factor-kappa b and inflammatory activation in human endothelial cells. J. Immunol. 157 (10), 4584–4590. doi: 10.4049/jimmunol.157.10.4584 8906837

[B82] Wooten R. M. Weis J. J. (2001). Host-pathogen interactions promoting inflammatory Lyme arthritis: Use of mouse models for dissection of disease processes. Curr. Opin. Microbiol. 4 (3), 274–279. doi: 10.1016/s1369-5274(00)00202-2 11378478

[B83] Yanai H. Ban T. Wang Z. Choi M. K. Kawamura T. Negishi H.. (2009). HMGB proteins function as universal sentinels for nucleic-acid-mediated innate immune responses. Nature 462 (7269), 99–103. doi: 10.1038/nature08512 19890330

[B84] Zhao E. Xu H. Wang L. Kryczek I. Wu K. Hu Y.. (2012). Bone marrow and the control of immunity. Cell Mol. Immunol. 9 (1), 11–19. doi: 10.1038/cmi.2011.47 22020068 PMC3251706

[B85] Zhong X. Lundberg M. Råberg L. (2020). Comparison of spleen transcriptomes of two wild rodent species reveals differences in the immune response against *Borrelia afzelii* . Eco.l Evol. 10 (13), 6421–6434. doi: 10.1002/ece3.6377 PMC7381583 32724523

[B86] Zhong X. Nouri M. Raberg L. (2019). Colonization and pathology of *Borrelia afzelii* in its natural hosts. Ticks Tick Borne Dis. 10 (4), 822–827. doi: 10.1016/j.ttbdis.2019.03.017 31005618

