# Supplementary figures and images for: Comparative transcriptome analysis of Peromyscus leucopus and C3H mice infected with the Lyme disease pathogen
Source: Front Cell Infect Microbiol. 2023 Apr 11;13:1115350. doi: 10.3389/fcimb.2023.1115350 (PMC10126474; doi:10.3389/fcimb.2023.1115350)

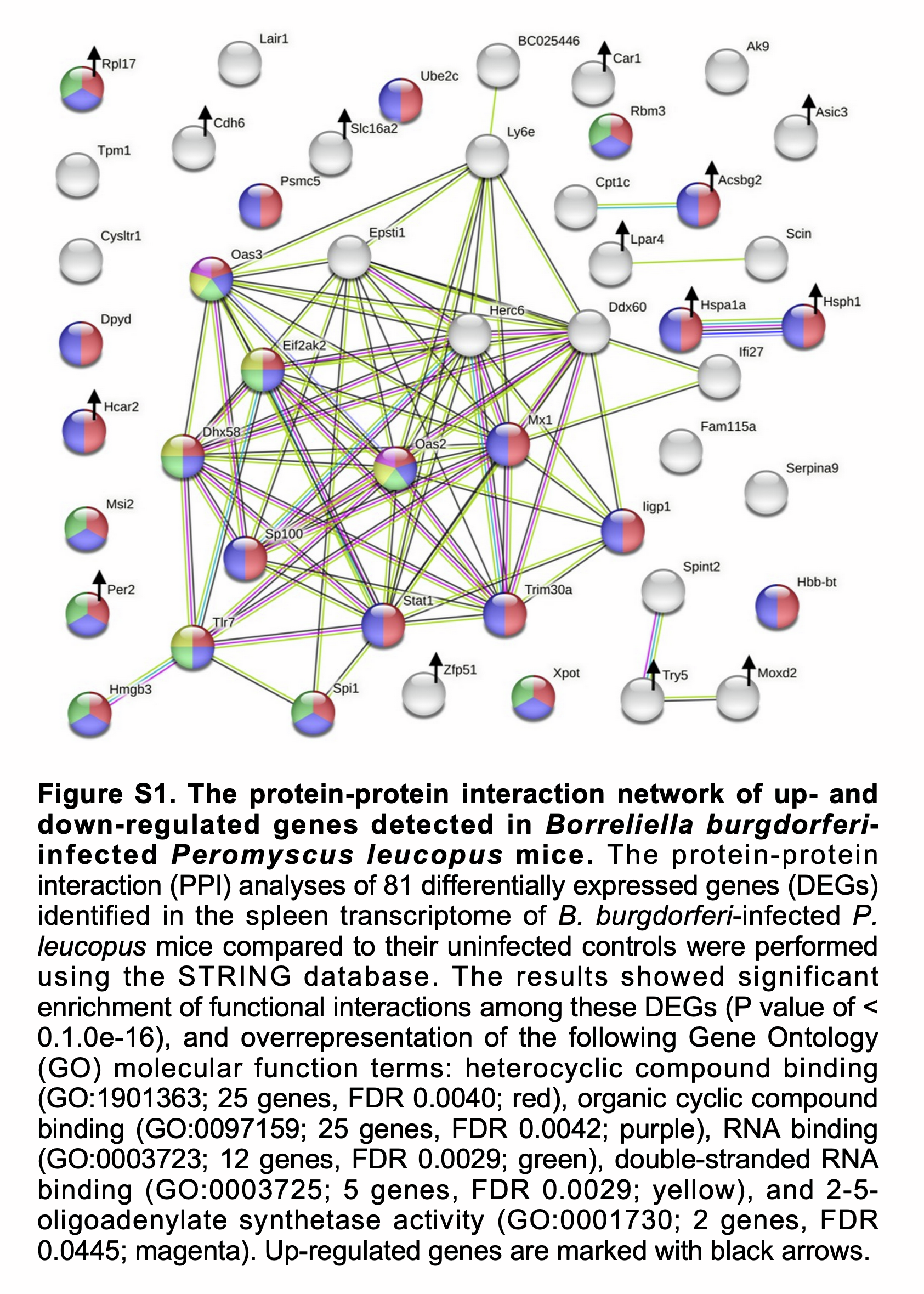

Supplement: Supplementary file 1 [file Image_1.tif]

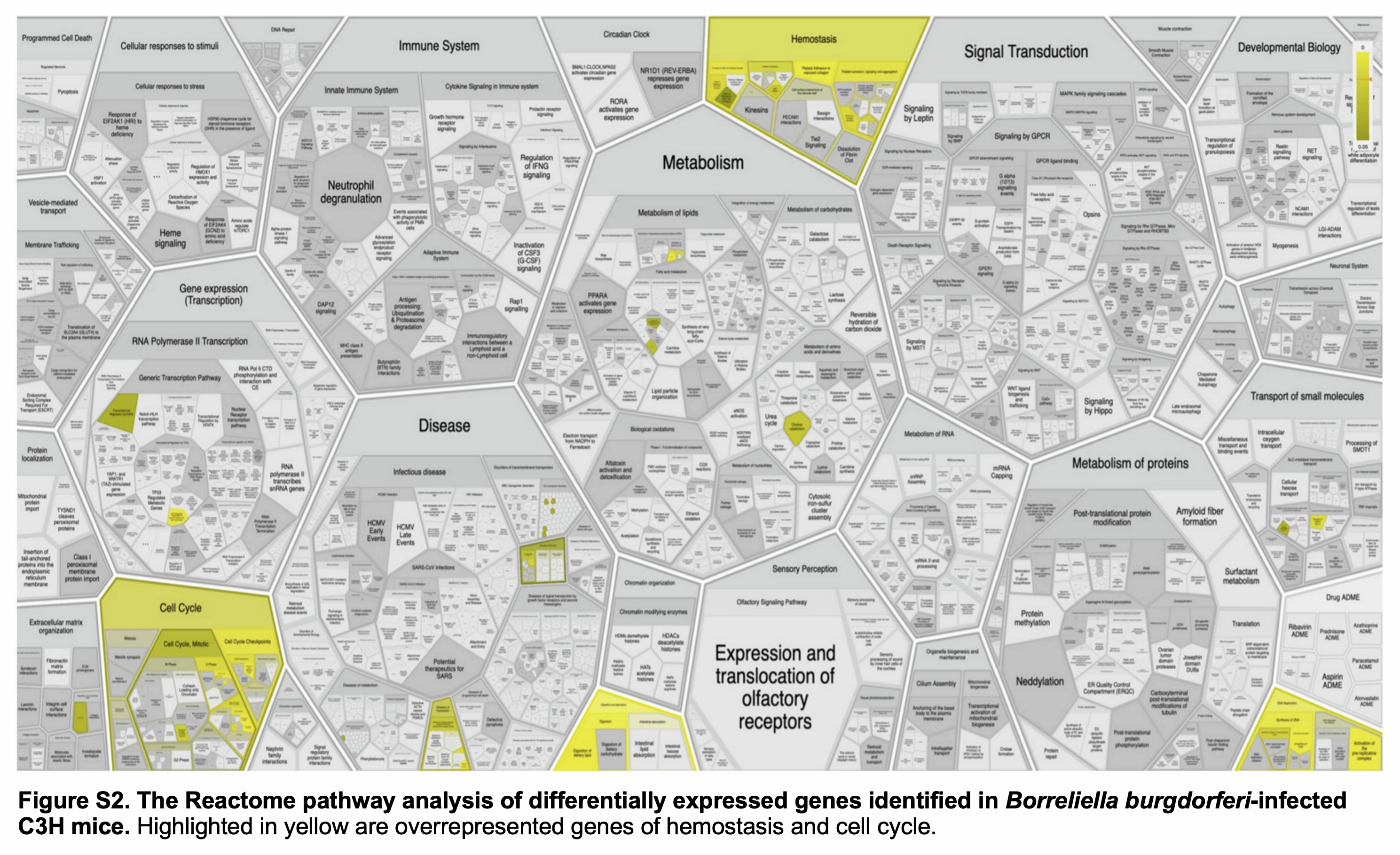

Supplement: Supplementary file 2 [file Image_2.tif]
