# Supplementary material for: Comparative transcriptome analysis of Peromyscus leucopus and C3H mice infected with the Lyme disease pathogen
Source: Front Cell Infect Microbiol. 2023 Apr 11;13:1115350. doi: 10.3389/fcimb.2023.1115350 (PMC10126474; doi:10.3389/fcimb.2023.1115350)
Supplement: Supplementary file 3 [file Table_1.docx]

**Table S1. Assessment of culture results of tissues collected from *Borreliella burgdorferi* strain 297-infected *Peromyscus leucopus* and C3H mice.**

| Tissue collected (day postinfection) | *P. leucopus* mice | C3H mice |
| --- | --- | --- |
| Blood (day 7) | 3/3* | 3/3 |
| Ear (day 21) | 3/3 | 3/3 |
| Ear (day 70) | 3/3 | 3/3 |
| Heart (day 70) | 3/3 | 3/3 |
| Bladder (day 70) | 3/3 | 3/3 |
| Tibiotarsal joint (day 70) | 3/3 | 3/3 |

*Values listed correspond to numbers of positive cultures/total number of tested samples.
